# Frailty is associated with susceptibility and severity of pneumonia in older adults (A JAGES multilevel cross-sectional study)

**DOI:** 10.1038/s41598-021-86854-3

**Published:** 2021-04-12

**Authors:** Kousuke Iwai-Saito, Yugo Shobugawa, Jun Aida, Katsunori Kondo

**Affiliations:** 1 Division of International Health, Graduate School of Medical and Dental Sciences, Niigata University, 1-757 Asahimachi-dori, Chuo Ward, Niigata, 951-8510 Japan; 2 Department of Active Aging (Donated By Tokamachi City, Niigata Japan), Graduate School of Medical and Dental Sciences, Niigata University, Niigata, Japan; 3 Department of Oral Health Promotion, Graduate School of Medical and Dental Sciences, Tokyo Medical and Dental University, 1-5-45 Yushima, Bunkyo City, Tokyo, 113-8549 Japan; 4 Liaison Center for Innovative Dentistry, Tohoku University Graduate School of Dentistry, 4-1 Seiryo-machi, Aoba Ward, Sendai City, Miyagi 980-8574 Japan; 5 Department of Social Preventive Medical Sciences, Center for Preventive Medical Sciences, Chiba University, Chuo-ku, Chiba, 260-8670 Japan; 6 Department of Gerontology and Evaluation Study, Center for Gerontology and Social Science, National Center for Geriatrics and Gerontology, 7-430 Morioka-cho, Obu, Aichi 474-8511 Japan

**Keywords:** Infectious diseases, Policy and public health in microbiology, Epidemiology

## Abstract

Pneumonia is a leading cause of mortality among older adults worldwide. Recently, several studies reported that frailty was associated with mortality among older adults hospitalized due to respiratory infectious diseases, including pneumonia. However, it is unknown whether frailty is associated with susceptibility to and severity of pneumonia in functionally-independent community-dwelling older adults. In this study, we examined whether frailty increased the susceptibility to pneumonia and hospitalization in older adults. We used cross-sectional data from the Japan Gerontological Evaluation Study; the data was collected by using mail-based, self-reported questionnaires from 177,991 functionally-independent community-dwelling older adults aged ≥ 65 years. Our results showed that frailty was significantly associated with both occurrence of and hospitalization due to pneumonia after adjustments with covariates; (Preference ratio {PR} 1.92, 95% confidence interval {95% CI} [1.66–2.22] and PR 1.80, 95% CI [1.42–2.28], respectively, *p* < 0.001 for the both). Pre-frailty was associated only with the occurrence of pneumonia. Besides, the instrumental activity of daily living, physical strength, nutrition status, oral function, homeboundness, and depression status in frail older adults were associated with either or both occurrence of and hospitalization due to pneumonia. Our results suggest that frailty influenced the susceptibility to and severity of pneumonia in older adults.

## Introduction

Pneumonia is a major cause of mortality and morbidity among older adults worldwide^1^. Community-acquired pneumonia (CAP) is a leading cause of morbidity among community-dwelling older adults in many countries and is different from hospital-acquired pneumonia (HAP) and ventilator-associated pneumonia (VAP). The overall incidence rates of CAP have been estimates as 1790–4000 in Japan, 2015; 630–1463 in the US, 2015, and 1400 in Europe, 2013 per 100,000 older adults aged ≥ 65 years^2–4^. A recent report showed that 6.8 million episodes of clinical pneumonia, including CAP, resulted in hospital admissions of older adults globally in 2015^5^.

Frailty is a state of increased vulnerability to stressors, including infectious diseases, which develops as a consequence of age‐related decline in multiple physiological and psychological systems, including the central nervous, endocrine, skeletal muscle, and immune systems^6,7^. A recent report showed that the pooled prevalence rates of frail older adults in 62 countries were 22–26%, based on the deficit accumulation model^8^. Several reports have recently shown that frailty was associated with increased severity and mortality of hospitalized older adults due to respiratory infectious diseases. Hewitt and colleagues showed that frailty in older adults hospitalized due to COVID-19 was associated with in-hospital mortality^9^. Lees and colleagues reported that the frailty of hospitalized older adults with influenza and acute respiratory illness was associated with lower odds of recovery^10^. Luo and colleagues recently reported that frailty was associated with the severity of CAP and mortality among hospitalized older adults^11^. However, it was unknown whether frailty was related to the susceptibility to and severity of pneumonia among community-dwelling older adults.

Therefore, we examined whether frailty was associated with susceptibility to pneumonia and hospitalization among functionally-independent community-dwelling older adults aged ≥ 65 years. We assessed associations between each physical or psychological condition in frailty and the prevalence and severity of pneumonia.

## Methods

### Study sample

The present study had a cross-sectional design and uses data from the Japan Gerontological Evaluation Study (JAGES). This project investigated the social determinants of health among non-institutionalized and functionally independent people aged 65 years or older, who did not receive benefits from public long-term care insurance (LTCI) between September 2016 and January 2017:

“The LTCI system was introduced in Japan in 2000 to address the demands of older persons with disabilities based on the concept of a user-oriented social insurance system with support for independence. Older people with a certification for LTCI service needs can utilize facility services, in-home services, and community-based services depending on their physical and cognitive impairments”^12^.

We used the data “JAGES 2016,” which was obtained from self-reported questionnaires mailed to and filled-in by community-dwelling individuals in 39 municipalities in 2016. In the JAGES 2016 wave, self-administered questionnaires were mailed to functionally independent adults aged 65 years or older who did not receive benefits from the LTCI insurance in Japan. The survey was conducted in the municipalities between September 2016 and January 2017. The data included 180,021 individuals who answered the questionnaires with the basic items (response rate was 70.2%). Participants who did not answer questions regarding age or sex were excluded (n = 2030). The data consisted of individuals who did not receive benefits from the LTCI on 1st April 2016. The data consisted of a three-stage hierarchal structure. The individual data were nested into 720 communities based on elementary or junior high school districts, and these communities were further nested into 39 municipalities.

### Outcome variables

The occurrence of pneumonia and hospitalization due to it in the past year from September 2016 to January 2017 were the outcome variables. The occurrence of pneumonia was assessed by asking “Did you fall sick in the past year?” and instructing the participants to select an appropriate answer from the following items: “Influenza,” “Pneumonia”, and “none of them.” Hospitalization due to pneumonia was assessed for participants who answered “Influenza” or “pneumonia” in the previous question by asking “If the sickness was due to influenza or pneumonia, were you hospitalized with relation to it?” and instructing the participants to select an answer from the following options: “Not hospitalized,” “Hospitalized due to influenza,” “Hospitalized due to pneumonia,” “Contracted influenza while I was hospitalized for other diseases,” and “Developed pneumonia while I was hospitalized for other diseases.” Only participants who answered “Hospitalized due to pneumonia” were considered to be hospitalized. The participants who answered “Influenza” in the first question and “Hospitalized due to pneumonia” in the second were included because influenza can cause primary viral or secondary bacterial pneumonia^13^.

### Frailty

Frailty was assessed using the Kihon Check List (KCL)^14^. KCL was developed by the Japanese Ministry of Health, Labor and Welfare to identify older adults requiring LTCI. KCL was included in the self-administered questionnaires of the JAGES 2016 wave. KCL consists of 25 questions classified into the following seven categories: instrumental activity of daily living (IADL), physical strength, nutritional status, oral function, homeboundness, cognitive function, and depressive mood (Supplemental Table 1). The scores from the KCL was well correlated with the validated assessments of physical strength, nutritional state, cognitive function, depressive mood, and the number of frailty phenotypes defined by the Cardiovascular Health Study criteria (CHS)^14^. Frailty was categorized into three groups based on KCL scores: robust, 0–3; pre-frail, 4–7; and frail, ≥ 8; scores were calculated from the KCL questions, which were validated with the pre-frail and frail categories established by the CHS criteria^14^. KCL variables were generated for each of the seven categories. Each KCL variable was categorized into three groups, based on answers to questions: 0, not applicable; 1, applicable; and 2 or ≥ 2 applicable.

### Covariates

Age was classified into two groups (65–74 years and ≥ 75 years). Educational attainment was categorized into five groups: < 6 years, 6–9 years, 10–12 years, ≥ 13 years, and others. Equivalized income was calculated by dividing the normalized household gross income in 2015 by the square root of the number of household members, and was categorized into five groups: < 0.5, 0.50–0.99, 1.00–1.99, 2.00–3.99, and ≥ 4.00 million yen. The household structure was assessed by asking the respondents the question, “Who do you live with?”. They were asked to choose from the following options: “no one,” “spouse,” “son,” “daughter,” “spouse of child,” “grandchild,” “brother or sister, “father,” “mother,” “father-in-law,” “mother-in-law,” and “other.” The responses were classified into six groups as follows: living alone, living with a spouse, living with children, living with a spouse and children, living in three-generation households (living with/without a spouse, but with one of the sons/daughters/son’s or daughter’s spouse and grandchildren), and living in a household structure other than the above five categories. Marital status was assessed by asking, “What is your marital status?” and participants were instructed to select one from the following five options: “Married,” “Widowed,” “Divorced,” “Never married” and “Other.” Smoking status was assessed by asking, “Do you smoke cigarettes?” and the participants were instructed to select appropriate answers from the following items: “Never smoked,” “Quit smoking ≥ 5 years ago”, “Quit smoking < 5 years ago,” “Smoke sometimes” and “Smoke almost every day.” The population density of the municipality was categorized as follows: metropolitan (density over 4000 people per km^2^), urban (density between 1,500 and 4000 people per km^2^), semi-urban (density between 1000 and 1499 people per km^2^), and rural (density below 1,000 people per km^2^)^15^. A municipality dummy variable was generated to adjust for differences in municipalities’ policies in preventing frailty^16^. The diabetes, respiratory, heart, kidney/prostate gland, hematological, or immune disease status was assessed by asking participants whether they were receiving any treatment or experiencing after-effects of any of the above diseases. Pneumococcal vaccination status was assessed by asking the participants, “Did you get a pneumococcal vaccination in the last five years? They chose from the following options: “No,” “Yes, I got vaccinated using my municipality's subsidy”, and “Yes, but I did not get vaccinated using my municipality's subsidy”. The participants who chose the two latter options were categorized as vaccinated.

### Statistical analysis

Multilevel Poisson regression analyses with random intercepts were performed to assess associations between frailty and pneumonia/hospitalization after adjusting for all the covariates. The data were structured in three levels: the individuals were nested within their elementary or junior high school districts and the districts were further nested within the municipalities. The covariates consist of all the covariates at individual-level and the municipality dummy variable used to adjust for differences in municipalities’ policies in older adult’s health including preventing frailty and pneumonia at municipality-level^16,17^. The individual-level covariates were: age group, sex, educational attainment, equivalized income, household structure, marital status, smoking status, municipality population density, diabetes, respiratory disease, heart disease, kidney/prostate gland disease, hematological/immune disease, and pneumococcal vaccination. The prevalence ratios (PRs) and 95% confidence intervals (95% CIs) were calculated after adjusting for all covariates. We used Stata version 14.2 (StataCorp., College Station, TX, USA) for all analyses, with a 2-tailed significance level set at 5%.

### Ethical consideration

The process of obtaining informed consent in the present study was as follows: the questionnaire was sent by mail with the explanation of the study; the participants read the written explanation about the purpose of study and replied. Hence, we considered that informed consent was provided by those who replied and sent back the questionnaire. The JAGES protocol in 2016 was approved by the ethics committee of National Center for Geriatrics and Gerontology (No. 992) and the ethics committee of Chiba University (No. 2493). We followed the STROBE Statement to report our observational study. This study was performed in accordance with the principles of the Declaration of Helsinki. Informed consent was obtained from all participants.

## Results

### The relationship between prevalence rates of pneumonia and hospitalization with frailty and other characteristics of older adults

Table 1 compares the prevalence rates of pneumonia in the past year with the characteristics of community-dwelling older adults. Frail and pre-frail older adults were 3- and 1.5-times, respectively, more likely to contract pneumonia than non-frail adults. Older adults, with ≥ 1 applicable question in KCL related to IADL, physical strength, nutrition status, oral function, homeboundness, cognitive function, and depression status, were likely to contract pneumonia than those with a score = 0 (none of the questions were applicable). Older adults aged ≥ 75 years, who were male, had lower education/income, who lived in three-generation households, had “other” marital status, who had quit smoking < 5 years ago, and who lived in rural or semi-urban areas were more likely to contract pneumonia than those in other categories. Older adults with diabetes, respiratory, heart, kidney/prostate gland, and hematological/immune diseases, and those who received pneumococcal vaccination were more likely to contract pneumonia more than those without any disease.

**Table 1:** Prevalence rates of pneumonia in comparison with the characteristics of older adults.

Variable	Category	Number	PR (%)	Variables	Category	Number	PR (%)
Frailty	Non-frail	46,103	1.0	Household structure (living alone or with family)	Alone	17,960	1.6
	Pre-frail	74,226	1.5		With spouse	76,735	1.7
	Frail	27,826	3.0		With children	13,484	1.7
IADL	0*	93,053	1.4		With spouse and children	25,782	1.6
	1	48,447	1.7		In a three-generation household	20,935	1.9
	≥ 2	30,470	2.3		Others	25,095	1.6
Physical strength	0	81,729	1.3	Marital status	Married	127,198	1.7
	1	50,748	1.7		Widowed	35,437	1.6
	≥ 2	38,648	2.4		Divorced	7995	1.7
Nutrition status	0	141,534	1.4		Never married	5272	1.3
	1	31,089	2.7		Others	1328	3.1
	2	2885	5.7	Smoking status	Never smoked	106,724	1.3
Oral function	0	93,575	1.2		Quit smoking ≥ 5 years ago	44,794	2.3
	1	50,797	1.8		Quit smoking < 5 years ago	5800	4.0
	≥ 2	30,043	2.8		Smoking sometimes	2821	2.0
Homeboundness	0	143,613	1.4		Smoking almost every day	16,230	1.2
	1	29,376	2.6	Municipality population density	Metropolitan	58,881	1.7
	2	3972	4.1		Urban	48,294	1.5
Cognitive function	0	115,270	1.5		Semi-urban	18,071	1.8
	1	44,923	1.9		Rural	54,745	1.8
	≥ 2	15,224	2.6	Diabetes	No	148,495	1.6
Depression status	0	99,421	1.2		Yes	22,991	2.1
	1	28,866	1.8	Respiratory disease	No	162,577	1.1
	≥ 2	37,415	2.6		Yes	8909	12.9
Age groups	65–74	101,025	1.4	Heart disease	No	154,312	1.6
	≥ 75	78,966	2.1		Yes	17,174	3.0
Sex	Male	82,257	2.1	Kidney/prostate gland disease	No	158,970	1.6
	Female	97,734	1.3		Yes	12,516	2.6
Educational attainment	> 6	1723	2.8	Hematological/immune disease	No	169,260	1.7
	6–9	57,731	1.8		Yes	2226	4.0
	10–12	72,100	1.6	Pneumococcal vaccination	No	94,509	1.1
	≥ 13	45,388	1.5		Yes	72,274	2.5
	The other	1117	2.1				
Equivalized income, million yen	< 0.5	6996	1.8				
	0.50–0.99	18,547	1.7				
	1.00–1.99	47,711	1.6				
	2.00–3.99	43,949	1.6				
	≥ 4.00	19,882	1.5				

*Numbers in IADL, physical strength, nutrition status, oral function, homebound, cognitive function, depressive mode are the numbers of the applicable KCL questions for the older adults.

*PR* prevalence rate, *IADL* instrumental activity of daily living, *KCL* Kihon checklist.

Table 2 compares the prevalence rates of hospitalization due to pneumonia with the characteristics of older adults. Frail and pre-frail older adults were 3.3- and 1.6-times, respectively, more likely to be hospitalized than non-frail adults. Older adults, with ≥ 1 applicable question in KCL from all the seven categories were more likely to be hospitalized than those with a score = 0 (none of the questions were applicable). Older adults aged ≥ 75, who were male, had lower education/income, who lived in three-generation households, had “other” marital status, who had quit smoking < 5 years ago, and who lived in rural areas were more likely to be hospitalized than those in other categories. Older adults with diabetes, respiratory, heart, kidney/prostate gland, and hematological/immune diseases and who received pneumococcal vaccination were more likely to be hospitalized than those without any disease.

**Table 2:** Prevalence rates of hospitalization due to pneumonia in comparison with the characteristics of older adults.

Variable	Category	Number	PR (%)	Variables	Category	Number	PR (%)
Frailty	Non-frail	1993	7.7	Household structure (living alone or with family)	Alone	784	14.9
	Pre-frail	3580	12.7		With spouse	3833	14.8
	Frail	1692	25.1		With children	579	13.6
IADL	0*	4483	11.4		With spouse and children	1220	14.8
	1	2352	15.3		In a three-generation household	1126	16.6
	≥ 2	1516	23.6		Others	1140	16.4
Physical strength	0	3701	11.1	Marital status	Married	6399	14.9
	1	2475	13.1		Widowed	1497	16.1
	≥ 2	2125	23.6		Divorced	397	13.9
Nutrition status	0	6498	12.2		Never married	217	11.1
	1	1777	22.0		Others	68	29.4
	2	223	38.6	Smoking status	Never smoked	4748	11.3
Oral function	0	3934	11.9		Quit smoking ≥ 5 years ago	2546	19.5
	1	2584	14.9		Quit smoking < 5 years ago	384	36.2
	≥ 2	1881	21.4		Smoking sometimes	155	19.4
Homeboundness	0	6733	12.2		Smoking almost every day	687	10.8
	1	1569	22.8	Population density	Metropolitan	2840	14.1
	2	249	41.4		Urban	2263	14.5
Cognitive function	0	5320	13.7		Semi-urban	926	14.9
	1	2284	16.0		Rural	2653	17.1
	≥ 2	883	21.2	Diabetes	No	7200	14.8
Depression status	0	4458	11.1		Yes	1178	19.2
	1	1453	14.3	Respiratory disease	No	6989	10.1
	≥ 2	2155	22.7		Yes	1389	42.1
Age groups	65–74	5020	10.4	Heart disease	No	7293	14.1
	≥ 75	3662	21.8		Yes	1085	24.3
Sex	Male	4402	19.3	Kidney/prostate gland disease	No	7608	14.7
	Female	4280	11.0		Yes	770	22.5
Educational attainment	> 6	76	32.9	Hematological/immune disease	No	8213	15.3
	6–9	2678	20.4		Yes	165	22.4
	10–12	3492	13.1	Pneumococcal vaccination	No	4296	10.5
	≥ 13	2296	11.8		Yes	4181	19.1
	The other	67	11.9				
Equivalized income, million yen	< 0.5	357	15.1				
	0.50–0.99	876	13.6				
	1.00–1.99	2294	14.5				
	2.00–3.99	2146	14.9				
	≥ 4.00	930	13.9				

*Numbers in IADL, physical strength, nutrition status, oral function, homebound, cognitive function, depressive mode are the numbers of the applicable KCL questions for the older adults.

*PR* prevalence rate, *IADL* instrumental activity of daily living, *KCL* Kihon checklist.

### Frailty was associated with susceptibility to and severity of pneumonia among community-dwelling older adults

**Figure 1:**
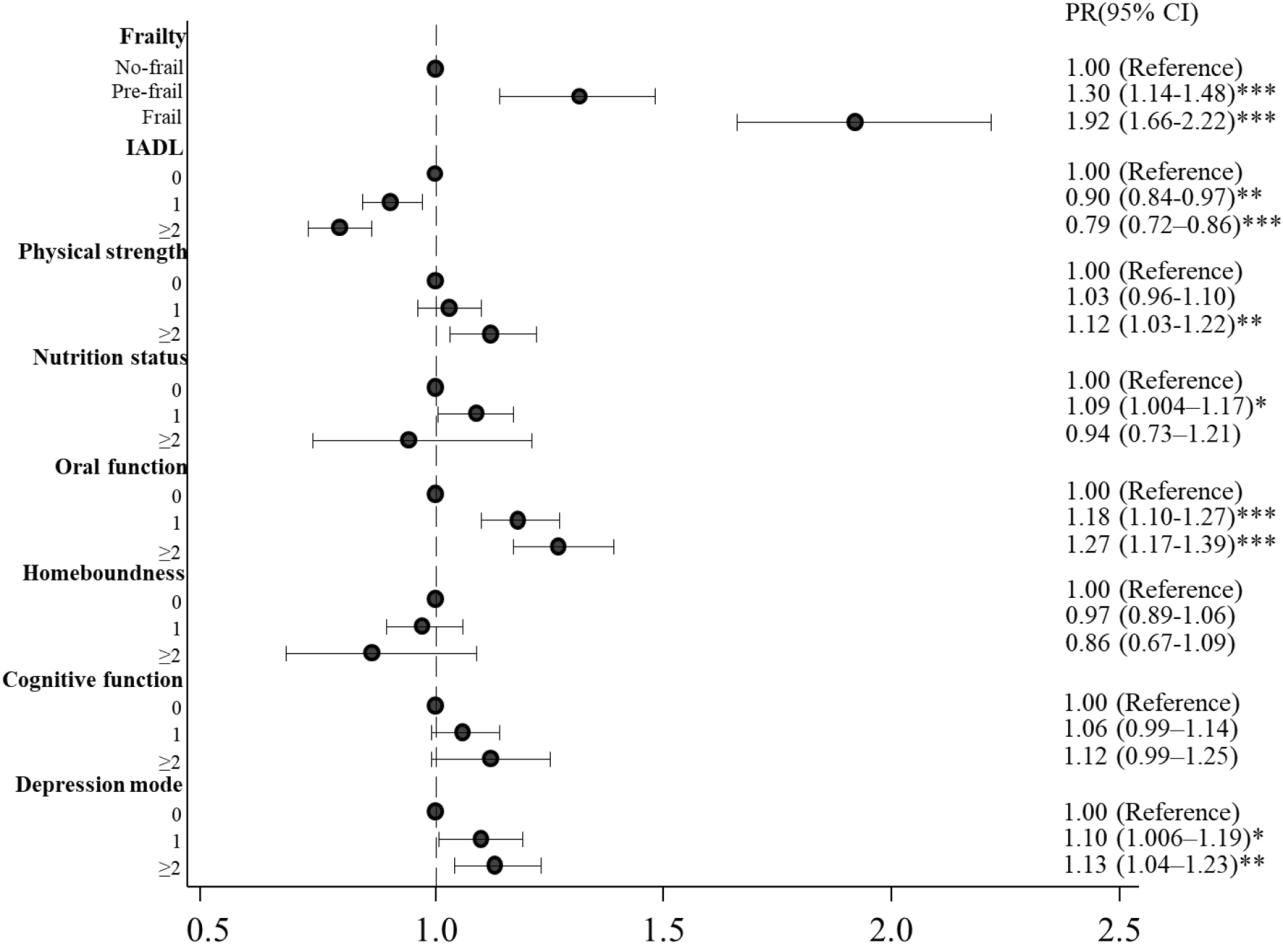
Prevalence ratios (PR) and 95% confidence intervals (95% CI) of associations between the occurrence of pneumonia and frailty or each of the Kihon checklist (KCL) categories in community-dwelling older adults. Numbers in KCL scores represent how many questions were applicable to older adults. *p < 0.05, **p < 0.01, ***p < 0.001. IADL, Instrumental Activity of Daily Living.

Figure 1 shows PR and 95% CI of the association between frailty and the occurrence of pneumonia in the past year among the community-dwelling older adults (upper side of the figure). After adjusting with all the covariates, both frailty and pre-frailty in older adults were significantly associated with the occurrence of pneumonia compared to non-frailty (PR 1.92 [95% CI 1.66–2.22] and PR 1.30 [95% CI 1.14–1.48], respectively, *p* < 0.001 for the both).

**Figure 2:**
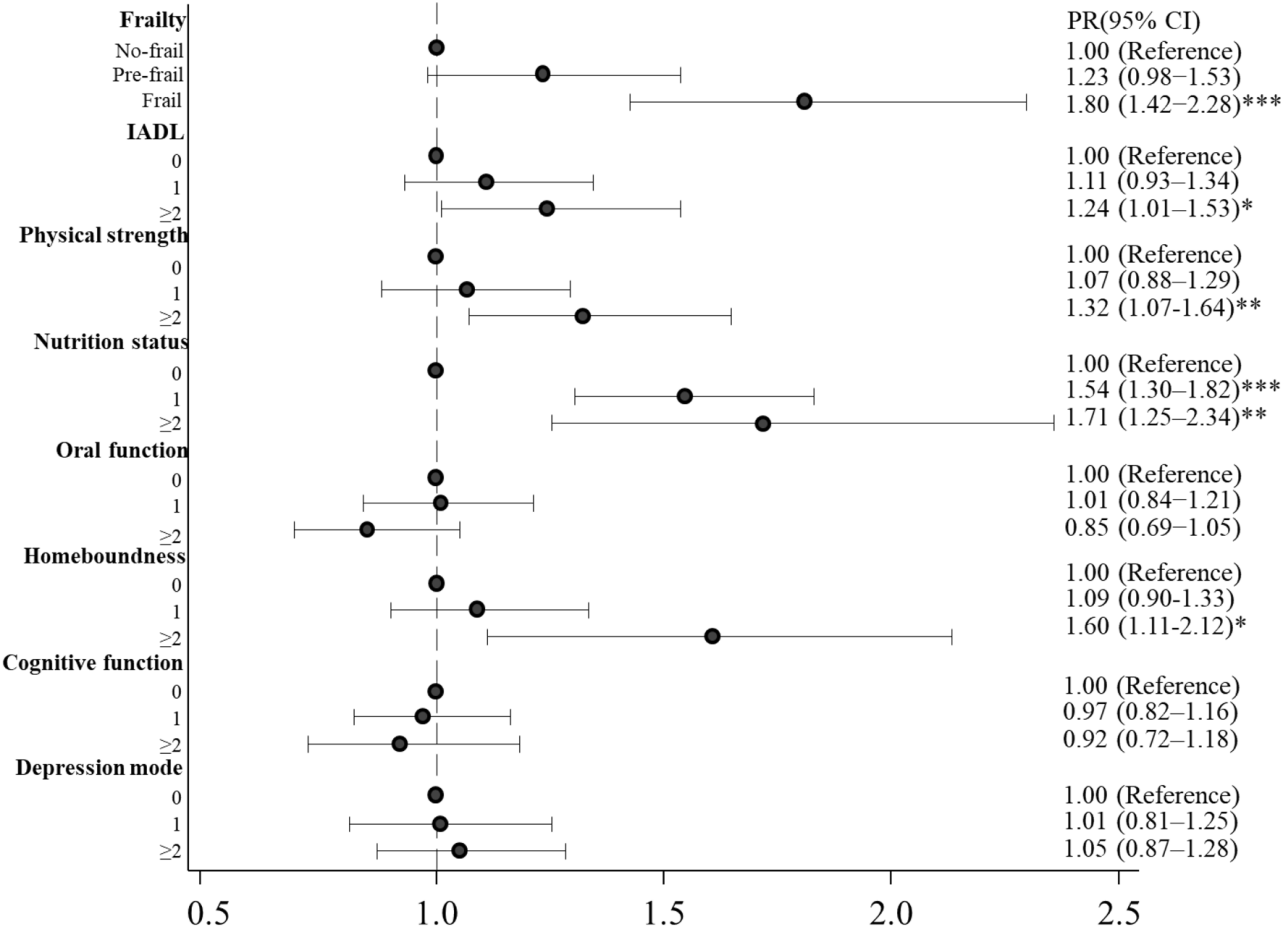
Prevalence ratios (PR) and 95% confidence intervals (95% CIs) of associations between the hospitalization due to pneumonia and frailty or each of the Kihon checklist (KCL) categories in community-dwelling older adults. Numbers in KCL scores represent how many questions were applicable to older adults. *p < 0.05, **p < 0.01, ***p < 0.001. IADL, Instrumental Activity of Daily Living.

Figure 2 shows PR and 95% CI of the association between frailty and the hospitalization due to pneumonia among the community-dwelling older adults (upper side of the figure). After adjusting with all the covariates, frailty in older adults was significantly associated with the hospitalization due to pneumonia compared to non-frailty (PR 1.80 [95% CI 1.42–2.28], *p* < 0.001). Pre-frailty was not significantly associated with hospitalization compared to non-frailty (PR 1.23 [95% CI 0.98–1.53]).

### Associations between each of the KCL categories and pneumonia/hospitalization among community-dwelling older adults

Figure 1 shows PRs and 95% CIs of associations between each of the KCL categories and the occurrence of pneumonia in the past year (from middle to bottom of the figure). After adjusting with all the other KCL categories and covariates, the lower or lowest IADL (1 or ≥ 2 KCL questions were applicable) was negatively and significantly associated with the occurrence of pneumonia (PR 0.90, 95% CI [0.84–0.97], *p* < 0.01 or PR 0.79, 95% CI [0.72–0.86], *p* < 0.001, respectively). The lowest physical strength was significantly associated with the occurrence of pneumonia in older adults (PR 1.12, 95% CI [1.03–1.22], *p* < 0.01). The lower nutrition status was significantly associated with the occurrence of pneumonia (PR 1.09, 95% CI [1.004–1.14], *p* < 0.05). The lower or lowest oral function was significantly associated with the occurrence of pneumonia in older adults (PR 1.18, 95% CI [1.10–1.27] or PR 1.27, 95% CI [1.17–1.39], respectively, *p* < 0.001 for the both). The worse and worst cases of depression mode were significantly associated with the occurrence of pneumonia in older adults (PR 1.10, 95% CI [1.006–1.19], *p* < 0.05 and PR 1.13, 95% CI [1.04–1.23], *p* < 0.01, respectively).

Figure 2 shows PRs and 95% CIs of associations between each of the KCL categories and the hospitalization due to pneumonia in the past year (from middle to bottom of the figure).

After adjusting with all the other KCL categories and covariates, the lowest IADL was significantly associated with the hospitalization due to pneumonia (PR 1.24, 95% CI [1.01–1.53], *p* < 0.05). The lowest physical strength was significantly associated with the hospitalization due to pneumonia in older adults (PR 1.32, 95% CI [1.07–1.64], *p* < 0.01). The lower or lowest nutrition status was significantly associated with the hospitalization due to pneumonia (PR 1.54, 95% CI [1.30–1.82], *p* < 0.001 or PR 1.71, 95% CI [1.25–2.34], *p* < 0.01, respectively). The worst homeboundness was significantly associated with the hospitalization due to pneumonia in older adults (PR 1.60, 95% CI [1.11 − 2.12], *p* < 0.05]).

## Discussion

We examined whether frailty was associated with the occurrence of and hospitalization due to pneumonia compared to non-frailty in community-dwelling older adults. Our analysis showed that frailty was significantly associated with both occurrence of and hospitalization due to pneumonia after adjusting for all covariates (Figs. 1 and 2) and that pre-frailty was significantly associated with the occurrence (Fig. 1). Besides, IADL, physical strength and nutrition status were significantly associated with the both occurrence of and hospitalization due to pneumonia, oral function and depression status were significantly associated with the occurrence, and homeboundness was significantly associated with the hospitalization (Figs. 1 and 2). Our results also suggested that frailty was associated with both susceptibility to and severity of pneumonia, and the six physical or psychological conditions in frailty were associated with either or both of them in community-dwelling older adults.

Several reports have recently shown that frailty was related to mortality in older adults hospitalized due to respiratory infectious diseases^9–11^. However, it was unknown whether frailty is associated with susceptibility to and severity of pneumonia in community-dwelling older adults. Our results showed that frailty was significantly associated with both occurrence of and hospitalization due to pneumonia in community-dwelling older adults, suggesting that frailty was associated with susceptibility to and severity of pneumonia in these adults (Figs. 1 and 2). Therefore, our results indicate the possibility that frailty may be one of the risk factors related to CAP and hospitalization in community-dwelling older adults. The pneumonia cases were likely CAP because HAP and VAP were excluded by asking corresponding questions to the participants (see “Outcome variables” in Methods). Our results also suggest that frailty may be an important indicator in the prevention of pneumonia among community-dwelling older adults, not only be an indicator of the mortality in older adults hospitalized for pneumonia as recently reported^11^.

Our results showed that low IADL was negatively and significantly associated with the occurrence of pneumonia after adjusting for all other KCL categories and covariates (Fig. 1). However, a negative association between low IADL and pneumonia has not been reported so far. Several reports have shown that community-dwelling older adults with low IADL were less social than those with normal IADL^18–20^. Social participation was reported to be associated with influenza among community-dwelling older adults^21^. Taken together, low IADL may present fewer opportunities for infection with respiratory infectious pathogens. However, our results showed that the lowest IADL was significantly associated with hospitalization among older adults (Fig. 2). Reichard and colleagues reported that adults with low IADL delayed or forewent receiving healthcare services twice more often than those with normal IADL, even if they were insured^22^. Older adults with the lowest IADL may have done the same when compared to those with normal IADL; they might have delayed visiting hospitals even if they had perceived subjective symptoms of pneumonia and hence, the pneumonia may have worsened and become severe leading to hospitalization.

The decline in immune function, so-called “immune senescence”, occurs in frail older adults; this is associated with the malfunction of the cellular and humoral immune systems^6,23^. Several research groups have reported that antibodies against influenza were more prevalent in physically active older adults than in sedentary individuals after vaccination; exercise enhanced the production of immunoglobulin A (IgA) secreted by the salivary gland in adults aged ≥ 65^24–26^. IgA is important for mucosal immunity as frontline protection against infections. Our results showed that the lowest physical strength was significantly associated with both occurrence of and hospitalization due to pneumonia (Figs. 1 and 2). Older adults with the lowest physical strength may be more susceptible to infections by pneumonia-causing pathogens due to decreased immunity, including the poor secretion of IgA; this may enable pathogens to invade the upper and lower respiratory tracts causing pneumonia and subsequent hospitalization.

Mitsutake and colleagues recently reported that socially-isolated and homebound older adults were significantly less likely to use outpatient and home medical care than those with neither characteristic, which may eventually lead to serious health problems that require more intensive treatment^27^. Our result showed that homeboundness was significantly associated with hospitalization due to pneumonia (Fig. 2). Older adults with homeboundness may not go to hospitals even if they recognize ills due to pneumonia. In addition, it may be difficult for physicians to have opportunities to diagnose pneumonia in those because they rarely visit hospitals. As a result, pneumonia in those with homeboundness may be severer and result in more hospitalizations. On the other hand, several reports have shown that homebound older adults were physically and psychologically unhealthy compared to those without homebound^28–30^. However, the physical and psychological conditions consisting of the IADL, physical strength, nutrition status, oral function, cognitive function and depression status were adjusted for the regression models to analyze the association between the hospitalization and homeboundness in our statistical analyses (Fig. 2). It suggests that there were the other reasons why the homeboundness was associated with the hospitalization among older adults than the physical or psychological frailty.

Several reports have already shown that poor nutrition is associated with CAP and hospitalization^31–33^. The decline in oral function, including poor swallowing and cough reflex, causes dysphagia, which induces aspiration pneumonia in frail older adults^4,34–37^. Geriatric depression is associated with the malfunction of the immune system and increased susceptibility to infection^38–40^. Our results showed that the nutrition status, oral function, and depression status were significantly associated with either or both occurrence of and hospitalization due to pneumonia (Figs. 1 and 2). The concordance between our results and the reported studies on the associations between the poor conditions and infectious diseases, including pneumonia, suggests that frailty is associated with susceptibility to pneumonia or hospitalization.

Our study has some limitations. First, this study has a cross-sectional design. Therefore, the possibility of pneumonia causing frailty cannot be completely excluded. However, the prevalence rate of pneumonia was 3.0% among all frail older adults (Table 1). Second, the occurrence of and hospitalization due to pneumonia were self-reported by the participants and not collected from medical records from the physicians in-charge. However, it is unlikely that they arbitrarily diagnosed themselves with pneumonia or non-medical professionals did so and they trusted it because most Japanese citizens have good medical access due to the national health coverage^41^. Third, we did not categorize the medical records based on the type of pneumonia, for example, CAP, HAP, or VAP. However, we expect most cases to be CAP because we excluded cases of nosocomial pneumonia with a question (see Methods). Besides, all the participants were functionally-independent community-dwelling older adults who did not receive benefits form the LTCI. Forth, it is possible that older adults with severe pneumonia could have been automatically excluded from the survey if the pneumonia was too severe to answer the questionnaire. Fifth, an information bias may have occurred in the survey for smoking if the participants did not honestly answer for their smoking status as self-reported-based assessments tend to underestimate smoking rates than the biological tests^42^. Therefore, our assessment might have counted the occurrence of pneumonia/hospitalization in the participants who did not honestly answer their smoking status despite actual smoking as pneumonia in those who never smoked or quit smoking (Tables 1 and 2). Sixth, a recall bias may have occurred in the survey for the pneumococcal vaccination. The question asked participants the vaccination status in the last five years, which they might not have been able to remember the vaccination if they had got it several years before the survey. Therefore, there may be participants who got the vaccination but did not remember the vaccination status during the survey (Tables 1 and 2). Besides, those who got the vaccination but contracted pneumonia could have been able to remember the vaccination status better than those who got the vaccination and did not contract pneumonia. As a result, a ratio of those who got the vaccination and contracted pneumonia may be overestimated (Tables 1 and 2).

## Conclusion

It was unknown whether frailty was associated with the susceptibility to and severity of pneumonia among community-dwelling older adults, although recent reports have shown that it is related to mortality due to respiratory infectious diseases in hospitalized older adults. We examined whether frailty was associated with the occurrence of and hospitalization due to pneumonia in functionally-independent community-dwelling older adults. Our results showed that frailty was significantly associated with both occurrence of and hospitalization due to pneumonia and that pre-frailty was significantly associated with the occurrence after adjusting for all covariates. Our results showed that six of all the seven physical and psychological frailty were significantly associated with either occurrence of, hospitalization due to pneumonia, or the both as follows: the oral function and depression mode were associated with the occurrence, the homeboundness was associated with the hospitalization, and IADL, physical strength and nutrition status were associated with the both. Our results suggested that frailty was associated with susceptibility to and severity of pneumonia, which is a leading cause of mortality and morbidity among community-dwelling older adults worldwide. This study showed frailty as a risk factor for increased susceptibility to and severity of pneumonia among community-dwelling older adults. It is necessary to assess whether frailty is also significantly associated with CAP diagnosed by medical doctors and the causal relationship between frailty and pneumonia should be confirmed in longitudinal studies in the future.

## Supplementary Information

Supplementary Information.

## Data Availability

All data used are from the JAGES study and are not third-party data. All enquiries are to be addressed at the JAGES data management committee via e-mail: dataadmin.ml@jages.net. All JAGES datasets have ethical or legal restrictions for public deposition due to inclusion of sensitive information from the human participants. Following the regulation of local governments which cooperated on our survey, the JAGES data management committee has imposed the restrictions upon the data.
