# Supplementary material for: Frailty is associated with susceptibility and severity of pneumonia in older adults (A JAGES multilevel cross-sectional study)
Source: Sci Rep. 2021 Apr 12;11:7966. doi: 10.1038/s41598-021-86854-3 (PMC8041848; doi:10.1038/s41598-021-86854-3)
Supplement: Supplementary file 1 — Supplementary Information. [file 41598_2021_86854_MOESM1_ESM.docx]

Frailty is associated with susceptibility and severity of pneumonia in older adults (A JAGES multilevel cross-sectional study).

Kousuke Iwai-Saito, Yugo Shobugawa, Jun Aida, and Katsunori Kondo

Supplemental Table S1. The Kihon check list

| No. | Questions | Answer |  |
| --- | --- | --- | --- |
| 1 | Do you go out by bus or train by yourself? | □0. YES | □1. NO |
| 2 | Do you go shopping to buy daily necessities by yourself? | □0. YES | □1. NO |
| 3 | Do you manage your own deposits and savings at the bank? | □0. YES | □1. NO |
| 4 | Do you sometimes visit your friends? | □0. YES | □1. NO |
| 5 | Do you turn to your family or friends for advice? | □0. YES | □1. NO |
| 6 | Do you normally climb stairs without using handrail or wall for support? | □0. YES | □1. NO |
| 7 | Do you normally stand up from a chair without any aid? | □0. YES | □1. NO |
| 8 | Do you normally walk continuously for 15 min? | □0. YES | □1. NO |
| 9 | Have you experienced a fall in the past year? | □1. YES | □0. NO |
| 10 | Do you have fear of falling while walking? | □1. YES | □0. NO |
| 11 | Have you lost 2 kg or more in the past 6 months? | □1. YES | □0. NO |
| 12 | Height: cm, weight: kg, BMI*: kg/m2 | □1. YES | □0. NO |
|  | If BMI is less than 18.5, this item is scored. |  |  |
| 13 | Do you have difficulties eating tough foods compared to 6 months ago? | □1. YES | □0. NO |
| 14 | Have you choked on your tea or soup recently? | □1. YES | □0. NO |
| 15 | Do you often experience a dry mouth? | □1. YES | □0. NO |
| 16 | Do you go out at least once a week? | □0. YES | □1. NO |
| 17 | Do you go out less frequently compared to last year? | □1. YES | □0. NO |
| 18 | Do your family or your friends point out your memory loss? | □1. YES | □0. NO |
|  | e.g. “You ask the same question over and over again.” |  |  |
| 19 | Do you make a call by looking up phone numbers? | □0. YES | □1. NO |
| 20 | Do you find yourself not knowing today’s date? | □1. YES | □0. NO |
| 21 | In the last 2 weeks, have you felt a lack of fulfilment in your daily life? | □1. YES | □0. NO |
| 22 | In the last 2 weeks, have you felt a lack of joy when doing the things you used to enjoy? | □1. YES | □0. NO |
| 23 | In the last 2 weeks, have you felt difficulty in doing what you could easily do before? | □1. YES | □0. NO |
| 24 | In the last 2 weeks, have you felt helpless? | □1. YES | □0. NO |
| 25 | In the last 2 weeks, have you felt tired without a reason? | □1. YES | □0. NO |

*BMI: Body Mass Index
